# Enhanced Osteocyte Differentiation: Cathepsin D and L Secretion by Human Adipose-Derived Mesenchymal Stem Cells

**DOI:** 10.3390/cells12242852

**Published:** 2023-12-17

**Authors:** Jung-Won Choi, Soyeon Lim, Seung Eun Jung, Seongtae Jeong, Hanbyeol Moon, Byeong-Wook Song, Il-Kwon Kim, Seahyoung Lee, Ki-Chul Hwang, Sang Woo Kim

**Affiliations:** 1 Medical Science Research Institute, College of Medicine, Catholic Kwandong University, Incheon Metropolitan City 22711, Republic of Korea; gardinia@hanmail.net (J.-W.C.); top98@naver.com (S.E.J.); 2 Department of Convergence Science, College of Medicine, Catholic Kwandong University, Incheon Metropolitan City 22711, Republic of Korea; slim724@cku.ac.kr (S.L.); songbw@ish.ac.kr (B.-W.S.); ilkwonkim@cku.ac.kr (I.-K.K.); sam1017@ish.ac.kr (S.L.); kchwang@cku.ac.kr (K.-C.H.); 3 The Interdisciplinary Graduate Program in Integrative Biotechnology, Yonsei University, Seoul 03722, Republic of Korea; 91seongtae@gmail.com; 4 Department of Integrated Omics for Biomedical Sciences, Graduate School, Yonsei University, Seoul 03722, Republic of Korea; moonstar3636@yonsei.ac.kr

**Keywords:** adipose-derived mesenchymal stem cells, hypoxic condition, osteoblast differentiation, two-dimensional electrophoresis, secretome

## Abstract

Adipose-derived mesenchymal stem cells (ASCs) have the potential to differentiate into bone, cartilage, fat, and neural cells and promote tissue regeneration and healing. It is known that they can have variable responses to hypoxic conditions. In the present study, we aimed to explore diverse changes in the cells and secretome of ASCs under a hypoxic environment over time and to present the possibility of ASCs as therapeutic agents from a different perspective. The expression differences of proteins between normoxic and hypoxic conditions (6, 12, or 24 h) were specifically investigated in human ASCs using 2-DE combined with MALDI-TOF MS analysis, and secreted proteins in ASC-derived conditioned media (ASC-derived CM) were examined by an adipokine array. In addition, genetic and/or proteomic interactions were assessed using a DAVID and miRNet functional annotation bioinformatics analysis. We found that 64 and 5 proteins were differentially expressed in hypoxic ASCs and in hypoxic ASC-derived CM, respectively. Moreover, 7 proteins among the 64 markedly changed spots in hypoxic ASCs were associated with bone-related diseases. We found that two proteins, cathepsin D (CTSD) and cathepsin L (CTSL), identified through an adipokine array independently exhibited significant efficacy in promoting osteocyte differentiation in bone-marrow-derived mesenchymal stem cells (BM-MSCs). This finding introduces a promising avenue for utilizing hypoxia-preconditioned ASC-derived CM as a potential therapeutic approach for bone-related diseases.

## 1. Introduction

Stem cell therapy is one of the most promising therapeutic strategies for tissue regeneration, and the method using mesenchymal stem cells (MSCs) has emerged as a prospective treatment [1]. MSCs have self-renewal ability and the potential to differentiate into chondrocytes, adipocytes, myoblasts, fibroblasts, and osteoblasts [2]. They can be largely isolated from the bone marrow and adipose tissue [3], and bone-marrow-derived MSCs (BM-MSCs) have been recognized as the most effective and reliable cell source for a long time despite the low yield of cells and limitations in clinical application [4]. BM-MSC-based therapies have poor survival in vivo after transplantation in hypoxic environments [5]. Adipose-derived MSCs (ASCs) are a new cell source for tissue engineering and regenerative medicine; they have recently received growing interest because adipose tissue is abundant and ASCs can be obtained easily and safely from donors and have potent regenerative capacity [6,7]. Adipose tissue is an endocrine organ derived from the mesoderm and is present everywhere in the body [8]. It plays a major role in maintaining energy metabolism by storing lipids and has multiple functions, including endocrine functions, glucose metabolism, and lipid metabolism [8,9]. ASCs can be used in various applications addressing osteoarthritis, heart disease, diabetes mellitus, and tissue reconstruction [6,8]. In addition to the most well-known sources of MSC, including BM, adipose tissue, and umbilical cord, MSC can be isolated from dental pulps, peripheral blood, placenta, muscle, skin, endometrium, and Wharton’s jelly [10].

Along with the strategy of using ASCs instead of BM-MSCs, there are additional strategies to increase the therapeutic effect and survival of ASCs, one of which is preconditioning [11]. Preconditioning of MSCs by hypoxia, pharmacological agents, chemical agents, cytokines, and physical factors is possible to sustain the survival and stemness of MSCs in their application for transplantation [12]. In preconditioning, the strategy is to expose ASCs to hypoxic environments before transplantation to mimic the oxygen environments in the body that are different from normal culture environments (21% O_2_) [13,14]. In fact, oxygen concentration varies among tissues, and oxygen tension in adipose tissue is very low [14]. The ASC responses to hypoxia vary according to the oxygen concentration (0.5–5% O_2_) in vitro [14]. Preconditioning using hypoxia may stimulate the secretion of growth factors, cytokines, and extracellular vesicles [15] and enhance cell survival, differentiation, and proliferation [16]. Likewise, low oxygen concentrations (0.5–5% O_2_) promote proliferation, migration, growth factor secretion, differentiation, and other physiological steps in ASCs [14]. Therefore, low oxygen tension must be considered to achieve more accurate and reliable experimental results because hypoxia preconditioning has significant advantages in the response of stem cells [17].

Cell differentiation by hypoxia preconditioning is critical in stem cell therapy because the differentiation time in the body is shortened after the implantation of stem cells. ASCs can differentiate into mesenchymal lineages, including adipogenic, osteogenic, and chondrogenic differentiation [18], and hypoxic conditions are an important factor for this differentiation [14]. Preconditioning of ASCs under low oxygen tension increases the adipogenic and osteogenic differentiation potential [19] and induces chondrogenic differentiation [20]. However, it has also been reported that hypoxia inhibits osteogenesis of ASCs [21], and this result is attributed to differences in experimental conditions. Osteoprogenitor cells are committed by the activation of master osteogenic transcriptional factors, such as runt-related transcription factor 2 (RUNX2), osterix (OSX), and drosophila distal-less 5 (DLX5). They then become a preosteoblast with early osteogenic genes, including alkaline phosphatase (ALP) and collagen1α1 chain (COL1A1), and the genes are kept in mature osteoblasts with other common osteoblast markers containing osteopontin (OPN), bone sialoprotein II (BSP II), and osteocalcin (OCN). The mature osteoblasts become bone lining cells or osteocyte or undergo apoptosis [22]. 

On the other hand, the therapeutic effect of MSCs is expected to be caused by paracrine signaling because the survival and differentiation of MSCs occur only in the lesion [23]. Some reports support this hypothesis; for example, Hocking AM and colleagues observed that many cell types responding to paracrine signaling from MSCs showed changes in cell responses, such as survival, proliferation, and migration [24]. In fact, MSC-conditioned medium (CM) containing paracrine-activity-mediated factors showed improved cardiac function [25], wound healing [26], liver regeneration [27], and bone regeneration [28] in animal models of different diseases.

In the present study, we aimed to study diverse changes in the cells and secretome of ASCs under a hypoxic environment over time and to present the possibility of ASCs as therapeutic agents from a different perspective. The expression differences of proteins between normoxic or hypoxic conditions (6, 12, or 24 h) were specifically investigated in human ASCs using 2-DE combined with a MALDI-TOF MS analysis, and secreted proteins in ASC-derived conditioned media (ASC-derived CM) were examined by an adipokine array. In our analysis, we identified several proteins with altered expression in hypoxic ASCs, many of which were associated with bone-related diseases according to a DisGeNET database search. Notably, cathepsin D (CTSD) and cathepsin L (CTSL) were among these proteins, which, through functional studies with BM-MSCs, were found to independently promote osteocyte differentiation. These findings support the potential application of ASC-derived conditioned media generated through hypoxia preconditioning as a promising therapeutic strategy for addressing bone-related diseases.

## 2. Materials and Methods

### 2.1. Cell Culture 

Human ASCs (Lonza, Walkerville, MD, USA) and BM-MSCs (Lonza) were cultured in 10% fetal bovine serum (FBS; HyClone, Logan, UT, USA)-supplemented Dulbecco’s modified Eagle’s medium (DMEM; HyClone) with 1% penicillin/streptomycin (HyClone) at a density of 5 × 10^4^ cells/cm^2^ in a 100 mm dish in a humidified atmosphere with 5% CO_2_ at 37 °C. Fourth-passage ASCs and BM-MSCs were used in this study. To induce osteocyte differentiation of BM-MSCs, we employed the StemPro osteogenesis differentiation kit (Gibco, Grand Island, NY, USA). BM-MSCs were seeded at a density of 5 × 10^3^ cells/cm^2^ in 12-well plates, and the complete osteogenesis differentiation medium was replenished every three days over a 21-day period with and without the addition of native human CTSD or CTSL proteins (Abcam, Cambridge, MA, USA). Throughout this experiment, we conducted specific assessments at various time points, including alkaline phosphatase assays at days 7, 10, and 14; alizarin red S staining at day 21; and gene expression analysis at day 21. 

### 2.2. Preparation of ASCs and ASC-Derived CM under Normoxic or Hypoxic Conditions

ASCs grown to 80% confluence were incubated under normoxic or hypoxic conditions for 6, 12, or 24 h after washing twice with serum-free media (SFM). For the hypoxic conditions, ASCs were incubated at 37 °C in 5% CO_2_, 5% H_2_, and 0.5% O_2_ in a chamber with an anaerobic atmosphere system (Technomart, Seoul, Republic of Korea). The cells were harvested after the 6, 12, or 24 h incubation period, and TRIZOL reagent (Life Technologies, Frederick, MD, USA) was added to the cells for two-dimensional electrophoresis (2-DE) analysis. The cell culture supernatants were collected separately to prepare CM. Cell debris was removed by two-step centrifugation at 480× *g* for 5 min and 2000× *g* for 10 min. The supernatant was concentrated to 1/50 using an Amicon Ultra centrifugal filter with a 3000 nominal molecular weight limit (EMD Millipore, Bedford, MA, USA) for subsequent experiments.

### 2.3. Two-Dimensional Electrophoresis (2-DE) Analysis

Proteins isolated from ASCs under normoxic or hypoxic conditions (6, 12, or 24 h) using TRIZOL reagent were dissolved in a rehydration buffer composed of 7 M urea (Sigma-Aldrich, St. Louis, MO, USA), 2 M thiourea (Sigma-Aldrich), 4% CHAPS (Sigma-Aldrich), 20 mM DTT (Sigma-Aldrich), 1 mM PMSF (Sigma-Aldrich), 2% IPG buffer (ampholyte 3/10, Bio-Rad, Hercules, CA, USA), and bromophenol blue (Sigma-Aldrich). The proteins (30 µg per gel) were permeated into gels of immobilized pH gradient (IPG) DryStrips (pH 3–10 and 18 cm; GE Healthcare, Buckinghamshire, UK), and the strips including proteins were rehydrated for 12 h in holders. IPG isoelectric focusing (IEF) of the strips was performed using an Ettan^TM^IPGphor^TM^ 3 system (GE Healthcare) according to the advanced-mode protocol: 1 h at 500 V, 3 h at 1000 V, and 6 h at 7000 V and then held at 7000 V until it reached 115 KVh. For the secondary separation of proteins, strips were electrophoretically applied to a 12% polyacrylamide gel using an Etthan DALTsix system (GE Healthcare). The gels, including separated proteins, were visualized using silver staining. The gels were imaged using a UMAX PowerLook 1120 system (Maxium Technologies, Akron, OH, USA), and the images were compared by group using modified ImageMaster 2D software V4.95 (GE Healthcare).

### 2.4. Peptide Mass Fingerprinting (PMF)

Spots were cut from gels and then digested with trypsin (Promega, Madison, WI, USA) and mixed with α-cyano-4-hydroxycinnamic acid (CHCA; Sigma-Aldrich) in 50% acetonitrile/0.1% trifluoroacetic acid (Sigma-Aldrich) for matrix-assisted laser desorption/ionization time-of-flight mass spectrometry (MALDI-TOF MS) analysis. Spectra were acquired using a Microflex LRF 20 (Bruker Daltonics, *m*/*z* 600–3000; 300 laser shots per spectrum), the peak list was generated using Flex Analysis 3.0, and internal calibration was performed using trypsin autodigested mass peaks (*m*/*z* 842.5099, 2211.1046). The threshold used for peak picking was 500 for the minimum resolution of monoisotopic mass, and the S/N threshold was 5. The most intense peaks were searched at a mass tolerance of 0.1 to 0.2 Da using MASCOT (http://www.matrixscience.com; Accessed on 6 January 2022), and the probability score for positive identification was determined using MOWSE (molecular weight search). The criteria for acceptance were as follows: −10*Log (*p*), where *p* is the probability that the observed match is a random event, and a score greater than 66 is significant (*p* < 0.05).

### 2.5. Functional Enrichment and Network Analysis

Identified proteins obtained from 2-DE/MALDI-TOF were analyzed using the database for annotation, visualization, and integrated discovery (DAVID). The analysis was performed using the complete DAVID Knowledgebase (Enterz Gene, UniProt, DisGeNET, KEGG, etc.) built upon the DAVID Gene concept, which combines multiple sources of functional annotations (https://david.ncifcrf.gov/; Accessed on 13 July 2022) [29]. We applied the network of each gene using the miRNet web database (https://www.mirnet.ca/; Accessed on 14 July 2022) [30]. It is a web-based platform designed to help network-based visualization of miRNA–target gene interactions coupled with improved functional analysis. 

### 2.6. Adipokine Array

The secretome of ASC-derived CM was investigated with a human adipokine array kit (R&D Systems (ARY024), Minneapolis, MN, USA) according to the manufacturer’s instructions. The intensity of each spot was quantified with modified ImageMaster 2D software V4.95, and the fold changes in the volume (%) in ASC-derived CM and hypoxic ASC-derived CM were calculated relative to the volume in medium and normoxic ASC-derived CM, respectively.

### 2.7. Mineralized Matrix Formation Assay

The cells were fixed in chilled 70% ethanol for 30 min at 4 °C and stained with 2% alizarin red S (Sigma-Aldrich) for 10 min at RT. The cells were observed under a microscope (CKX41; Olympus, Tokyo, Japan) and a digital camera (eXcope T300; Olympus).

### 2.8. Alkaline Phosphatase Assay

The alkaline phosphatase activity in the conditioned media of osteocyte-like cells (OLC) was assessed using an alkaline phosphatase assay kit (Abcam) following the manufacturer’s instructions. ALP activity was calculated as follows: ALP activity = ((B/(TxV))xD), where B represents the quantity of *p*NP in the sample well determined from a standard curve (in µmol), T stands for the reaction time (in minutes), V is the original sample volume added to the reaction well (in mL), and D is the sample dilution factor.

### 2.9. RNA Isolation, Reverse Transcription (RT), and Quantitative Real-Time RT-PCR (qRT-PCR)

Total RNA was isolated from cells using an easy-spin total RNA extraction kit (iNtRON Biotechnology, Seongnam, Republic of Korea), and oligo (dT)-primed cDNA was synthesized from the total RNA using the Maxime RT PreMix kit (iNtRON Biotechnology) according to the manufacturer’s instructions. Quantitative real-time PCRs were carried out using the StepOnePlus real-time PCR system (Thermo Fisher Scientific, Waltham, MA, USA) with the SYBR Green Dye system (SYBR Premix Ex Taq (Tli RNase Plus) and ROX reference dye (TAKARA Bio INC., Foster City, CA, USA)). The transcript level of each gene was normalized to the *GAPDH* transcript levels. Primers were designed using Primer3 and BLAST, and the primer set sequences are listed in Table 1.

### 2.10. Statistical Analysis

All data were compared and analyzed using one-way analysis of variance (ANOVA) with the Statistical Package of Social Science (SPSS, version 14.0K). The results of the experiment are presented as the mean ± SEM. When ANOVA indicated a significant effect, the group average showed a significant difference at *p* < 0.05 based on the protected least-significant difference (LSD) test (*p* < 0.05).

## 3. Results

### 3.1. Differentially Expressed Proteins in ASCs under Hypoxic Stress

**Figure 1:**
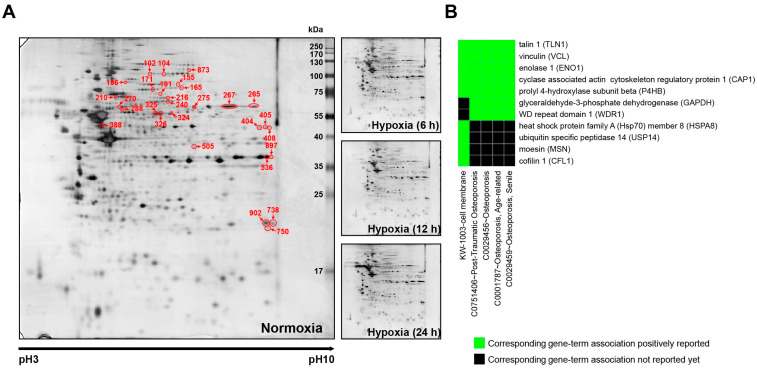
Discovery of oxygen-tension-related factors in ASCs using proteomic analyses. (**A**) Representative silver-stained 2-DE gel images. (**B**) Corresponding gene-term association in 2D view of DAVID functional annotation clustering. The 2-DE experiments were performed in triplicate for each individual.

**Figure 2:**
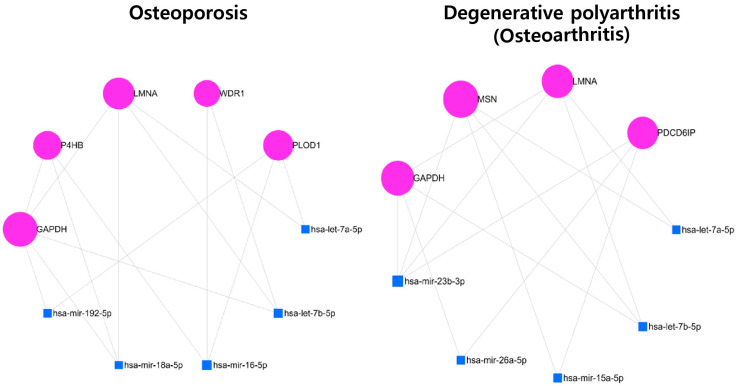
Bone-related disease prediction of up- and downregulated oxygen-tension-related factors in ASCs from miRNet target prediction in a DisGeNET database.

The 2-DE-based proteomic experiments were performed to observe changes in protein expression over time under hypoxic conditions in human ASCs. Proteins were separated into two steps using pH 3–10 IEF strips and 12% (*w*/*v*) SDS-PAGE. Approximately 700 individual spots with pH values between 3 and 10 and molecular weights between 6 and 240 kDa were detected (Figure 1). Among the 700 individual spots, 84 spots were significantly regulated in ASCs in a hypoxic environment, and 64 spots were identified using peptide mass fingerprinting (PMF) with matrix-assisted laser desorption/ionization (MALDI) time-of-flight (TOF) mass spectrometry (MS) (Figure 1A, Table 2, Supplementary Figure S1, and Supplementary Table S1). We carried out network analysis using the gene names of 64 proteins for DAVID functional annotation tools. The functional classification tool lists genes into functionally related groups to help unravel the biological content in gene functional classification. Gene functional classification results show gene members and their associated annotation terms in a 2D view. The highest enrichment scores include those for osteoporosis, posttraumatic osteoporosis, and cell membrane functions. Therefore, we discovered that seven genes (*TLN1*, *VCL*, ENO1, *CAP1*, *P4HB*, *GAPDH*, and *WDR1*) were related to osteoporosis by the DisGeNet database, and nine genes (*TLN1*, *VCL*, *ENO1*, *CAP1*, *P4HB*, *HSPA8*, *USP14*, *MSN*, and *CFL1*) were associated with the cell membrane by UniProtKB_Cellular Components (Figure 1B). In addition, target interaction network analysis by network-based visual analysis (miRNet) showed that five genes (*GAPDH*, *PLOD1*, *LMNA*, *WDR1*, and *P4HB*) with five miRNAs (hsa-let-7a/b-5p, hsa-let-7b-5p, hsa-mir-16-5p, hsa-mir-18a-5p, and hsa-mir-192-5p) were associated with osteoporosis by DisGeNET (Figure 2). Four target genes (*LMNA*, *MSN*, *PDCD6IP*, and *GAPDH*) with five miRNAs (hsa-let-7a-5p, hsa-let-7b-5p, hsa-mir-15a-5p, hsa-mir-26a-5p, and hsa-mir-23b-3p) were associated with the pathway in osteoarthritis according to the results of the miRNet network analysis.

### 3.2. Differentially Expressed Proteins by Hypoxic Stress in ASC-Derived CM

**Figure 3:**
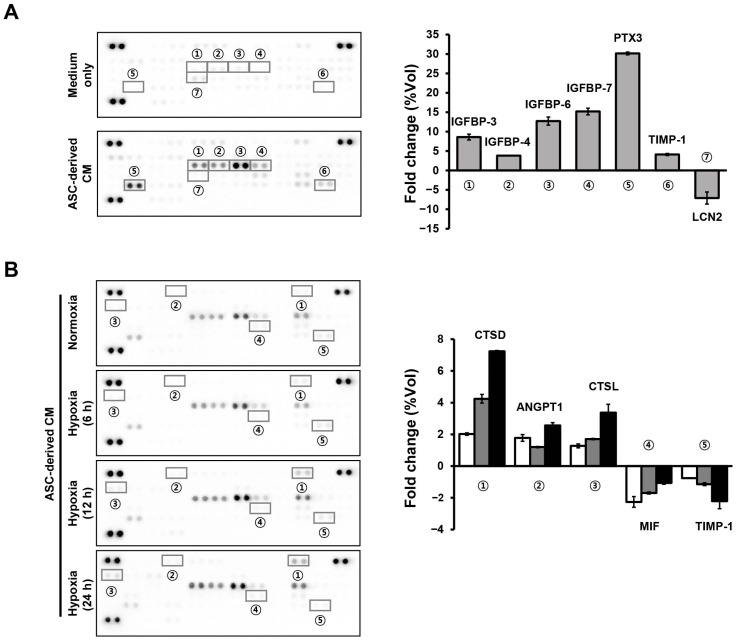
Identification of ASC-derived CM-enriched cytokines and cytokines with differential expression by oxygen tension in ASCs. Cytokines that were increased or decreased more than two-fold in ASC-derived CM compared to cell-free medium (**A**) and in hypoxic ASC-derived CM compared to normoxic ASC-derived CM (**B**) in experiments using an adipokine array kit. The same number with a circle in the array map and bar graph means the same protein.

In addition to the changes in protein expression in ASCs under low oxygen tension, ASC-derived CM containing secreted proteins was also investigated using an adipokine array that can detect 58 different cytokines (Supplementary Table S2). Above all, the expression levels of CM-derived cytokines were compared to those of the seven detected CM-derived adipokines (insulin-like growth factor-binding protein (IGFBP)-3, IGFBP-4, IGFBP-6, IGFBP-7, pentraxin (PTX) 3, TIMP metallopeptidase inhibitor (TIMP)-1, and lipocalin (LCN) 2), which were more than twice or less than twice as expressed as CM-derived cytokines (Figure 3A). Moreover, the expression levels of hypoxic CM-derived adipokines (6, 12, or 24 h) were compared to normoxic CM-derived adipokines individually, and five adipokines (cathepsin (CTS) D, angiopoietin (ANGPT) 1, CTSL, macrophage migration inhibitory factor (MIF), and TIMP-1) were found to have increased or decreased more than twice as much as the normoxic CM-derived adipokines (Figure 3B).

### 3.3. Effects of Cathepsin D (CTSD) and Cathepsin L (CTSL) in Osteogenic Lineage Differentiation of BM-MSCs

**Figure 4:**
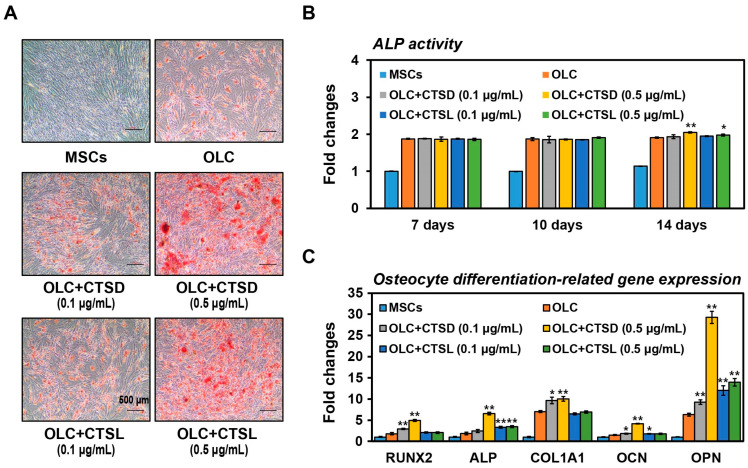
Impact of cathepsin D (CTSD) and cathepsin L (CTSL) on osteogenic lineage differentiation of BM-MSCs. (**A**) Alizarin red staining of BM-MSCs-derived osteocyte-like cells (OLCs) at 21 days, cultured under differentiation conditions with and without CTSD or CTSL. Representative images were captured at 40x magnification, and scale bars represent 500 μm. (**B**) Measurement of alkaline phosphatase (ALP) activity in conditioned medium (CM) of BM-MSCs-derived OLCs with and without CTSD or CTSL at 7, 10, and 14 days under differentiation conditions using an ALP activity assay. Activity values were calculated as described in the Materials and Methods section and then normalized to the OLC group (control group). (**C**) Quantification of mRNA expression levels of osteocyte-differentiation-related genes in BM-MSCs-derived OLCs with and without CTSD or CTSL at 21 days under differentiation conditions using qRT-PCR analysis. qRT-PCR values are presented as the normalized target gene expression levels relative to the GAPDH transcript levels (2^−ΔΔCt^) and further normalized to the OLC group (control group). Significant differences between OLC group and other groups were determined via ANOVA, with *p* values indicated as * *p* < 0.05 and ** *p* < 0.01.

Building on the insights gained from network analysis (Figure 2) and adipokine array results (Figure 3), in vitro experiments were conducted using bone-marrow-derived mesenchymal stem cells (BM-MSCs). The BM-MSCs were cultured in osteogenic differentiation medium for a duration of 21 days with and without the presence of cathepsin D (CTSD) or cathepsin L (CTSL) proteins introduced at 3-day intervals. Our study investigated various aspects of osteocyte differentiation, including matrix mineralization, alkaline phosphatase (ALP) activity, and the expression of osteocyte-differentiation-related genes as indicative characteristics (Figure 4). Under osteocyte differentiation conditions, the cells exhibited pronounced red coloration, and the addition of CTSD and CTSL independently heightened the intensity of alizarin red S staining compared to the control cells (Figure 4A). In other words, CTSD and CTSL fostered greater matrix mineralization in BM-MSCs within the osteogenic differentiation medium. Furthermore, the presence of CTSD and CTSL separately elevated ALP activity at a specific concentration in BM-MSCs on the 14th day of the osteocyte differentiation process (Figure 4B). Beyond matrix mineralization and ALP activity in BM-MSCs, we explored alterations in the expression of osteocyte-differentiation-related markers in BM-MSCs differentiating into OLCs, both with and without CTSD or CTSL, using qRT-PCR analysis (Figure 4C). CTSD induced a more significant increase in the gene expression of RUNX2, ALP, COL1A1, and OCN, whereas CTSL led to an increment in the expression of ALP, OCN, and OPN (see Figure 4C). Based on the findings presented in Figure 4, it is evident that CTSD and CTSL may independently facilitate differentiation into osteogenic lineages within BM-MSCs under an osteocyte differentiation environment.

## 4. Discussion

We found 64 spots that showed significant expression changes in human ASCs under hypoxic conditions using 2-DE combined with MALDI-TOF MS analysis (Figure 1 and Table 2). In addition, DisGeNET database analysis of gene–disease associations showed seven genes (*TLN1*, *VCL*, *ENO1*, *CAP1*, *P4HB*, *GAPDH*, and *WDR1*) in the functional annotation clustering of DAVID informatics and seven genes (*GAPDH*, *P4HB*, *LMNA*, *WDR1*, *PLOD1*, *MSN*, and *PDCK6IP*) in the miRNet analytics platform among the 64 spots associated with bone-related diseases, such as osteoarthritis and osteoporosis (Figure 1B and Figure 2). With comprehensive functional annotation, preconditioning ASCs under low oxygen tension improved the osteogenic differentiation potential [19,31], but there were also contradictory consequences [21,32]. These results suggest that the hypoxic environment of ASCs can stimulate the secretion of osteogenic-differentiation-related factors and directly affect the osteogenic differentiation of cells. Therefore, we analyzed the secretome in hypoxic ASC-derived CM (Figure 3), and osteocyte differentiation of the detected proteins was investigated (Figure 4).

We discovered three adipokines that were increased by hypoxic time in hypoxic ASC-derived CM using an adipokine array: CTSD, ANGPT1, and CTSL (Figure 3). These adipokines may be associated with the effects of hypoxic ASC-derived CM alone or together on the differentiation of osteoblast-like cells into osteocytes. Cathepsins primarily play a role in lysosomal protein turnover and are involved in extensive physiological processes, including antigen presentation, bone remodeling, and epidermal homeostasis [33]. They are mainly expressed in osteoclasts and are involved in bone remodeling and resorption [34]. Bone remodeling in osteoblasts and osteoclasts is associated with cathepsins [35], and CTSK in particular contributes to matrix maintenance and recycling of collagen I in osteoblasts [36]. However, few reports have reported the functions of other forms of cathepsins, including CTSD and CTSL, in osteoblasts. It can be inferred that CTSD and/or CTSL may also be involved in matrix mineralization of osteogenic differentiation. ANGPT1, ANGPT2, and vascular endothelial growth factor can regulate angiogenesis at the site of ossification and bone turnover in growing human bones [37]. In particular, ANGPT1 increases bone mass, induces osteogenesis, and modulates bone remodeling though the regulation of angiogenesis in osteoblasts [38,39]. In line with earlier observations, increased ANGPT1 was measured in hypoxic ASC-derived CM (Figure 3), suggesting ANGPT1 may have contributed to the osteoblast-differentiation-induced effects of hypoxic ASC-derived CM. 

Limited information is available regarding the bone-related functions of cathepsin D (CTSD) and cathepsin L (CTSL). In our study, we specifically chose to investigate these two proteins among the proteins detected in the adipokine array for in vitro functional analysis. While the potential roles of CTSD and CTSL in osteocyte differentiation were explored, it is worth noting that we conducted these experiments in BM-MSCs as they demonstrated a more favorable response to osteocyte differentiation compared to ASCs despite the diverse range of differentiation potential [40]. Our functional study revealed that both CTSD and CTSL independently promoted osteocyte differentiation in BM-MSCs under differentiation conditions (see Figure 4). Notably, we observed subtle differences in the extent to which CTSD and CTSL influenced osteocyte differentiation, as evidenced by variations in mineralization and ALP activity (as shown in Figure 4A,B). In particular, CTSD exhibited an enhanced induction of gene expression for RUNX2, ALP, COL1A1, and OCN, whereas CTSL primarily led to an increase in ALP, OCN, and OPN expression (refer to Figure 4C). These distinctions in their impact on osteocyte differentiation are likely attributed to these nuanced differences. 

While the association between cathepsin D (CTSD) and cathepsin L (CTSL) and osteogenic/chondrogenic differentiation is not well established, there is existing literature highlighting their correlation with adipogenic differentiation. Notably, CTSD expression is upregulated in the adipose tissues of obese individuals and is increased during adipogenic differentiation to regulate adipogenesis [41,42]. Similarly, elevated levels of CTSL have been reported in obese and diabetic patients, influencing adipogenesis and glucose tolerance [43]. Despite the limited number of reports on this topic, consideration of the role of CTSD and CTSL in adipogenic differentiation has the potential to add considerable depth and comprehensiveness to our study. 

Hypoxic conditions can affect the differentiation of ASCs into mesenchymal lineages, including osteogenic differentiation, although contradictory results have been reported under experimental conditions [14,19,21]. These results suggest that ASC-derived CM under hypoxic conditions may contain osteogenic-differentiation-related factors and can affect the osteogenic differentiation of other cell types directly and/or indirectly. In fact, it has been reported that the paracrine effect of MSCs has an impact on osteogenic differentiation and bone regeneration without preconditioning [28,44,45]. Importantly, this study represents the first instance demonstrating that hypoxic ASC-derived conditioned media enriched with CTSD and CTSL can autonomously promote the differentiation of BM-MSCs into osteogenic lineages. These findings bear considerable significance as they underscore the potential of utilizing hypoxic ASC-derived conditioned media in the treatment of bone-related diseases, particularly due to their impactful effects on osteogenic differentiation. When preconditioned ASCs under a hypoxic environment are used for clinical treatment in bone-related diseases, CM can be used together or alone to increase the effectiveness of treatment. The CM has the advantages of being easy to store, transport, and handle [45]. Therefore, therapeutic agents using hypoxic ASC-derived CM may have a high therapeutic effect at low cost on bone-related diseases. 

## 5. Conclusions

In our investigation, we identified 64 distinct protein spots exhibiting significant expression alterations in human ASCs subjected to hypoxic conditions by employing a combination of 2D gel electrophoresis (2-DE) and MALDI-TOF MS analysis. Further functional analysis utilizing the DAVID and miRNet analytics platforms revealed multiple genes associated with pathways linked to bone-related diseases. In our functional studies, two proteins, CTSD and CTSL, identified through adipokine array analysis were shown to independently and effectively enhance osteocyte differentiation in BM-MSCs. This discovery introduces a novel avenue for the potential therapeutic application of ASC-derived conditioned media (CM) obtained through hypoxia preconditioning, thereby offering a promising treatment option for bone-related diseases. Notably, our study marks the first report of the enhanced osteogenic differentiation potential of hypoxic ASC-derived CM enriched with CTSD and CTSL.

## Figures and Tables

**Table 1:** Sequences of primers used for qRT-PCR.

**Genes**		**Primer Sequence (5′-3′)**
*ALP*	F ^(a)^ R ^(b)^	GACCCTTGACCCCCACAAT CGCCTCGTACTGCATGTCCCCT
*COL1A1*	F R	CCGGAAACAGACAAGCAACCCAAA AAAGGAGCAGAAAGGGCAGCATTG
*RUNX2*	F R	AAGGGTCCACTCTGGCTTTG CTAGGCGCATTTCAGGTGCT
*OCN*	F R	TCACACTCCTCGCCCTATT TGAAAGCCGATGTGGTCAG
*OPN*	F R	CATATGATGGCCGAGGTGAT CATCCAGCTGACTCGTTTCA
*GAPDH*	F R	CATGGGTGTGAACCATGAGA GGTCATGAGTCCTTCCACGA

^(a)^ F, sequence from sense strands; ^(b)^ R, sequence from antisense strands.

**Table 2:** List of identified proteins showing differential expression in ASCs according to the exposure time of the hypoxic environment.

Spot ID ^(a)^	Gene Name	Description	Acc. No. ^(b)^	Nominal Mass (Mr) ^(c)^	Calculated PI	Score ^(d)^	Fold Change		
							HPX6/NMX ^(e)^	HPX12/NMX	HPX24/NMX
102	PDCD6IP	Programmed cell death 6-interacting protein isoform 1	gi|22027538	96,590	6.13	139	−1.46	−1.74	−1.12
104	EEF2	Elongation factor 2	gi|4503483	95,277	6.41	136	1.97	1.73	1.44
136	XRCC5	X-ray repair cross-complementing protein 5	gi|10863945	83,222	5.55	128	−1.92	−1.70	−1.84
191	XRCC6	X-ray repair cross-complementing protein 6 isoform 3	gi|573014819	64,528	9.32	157	2.00	1.32	1.32
155	PLOD1	Procollagen-lysine 1,2-oxoglutarate 5-dioxygenase 1	gi|16741721	84,114	6.47	138	1.93	1.22	1.49
165	LMNA	Lamin A/C transcript variant 1	gi|57014043	74,322	6.73	206	1.57	−1.00	−1.18
171	MSN	Moesin	gi|4505257	67,892	6.08	216	1.60	−1.03	−1.15
210	HSPA1A	HSP70-2	gi|4529892	70,267	5.48	181	1.90	1.11	−1.25
216	WDR1	WD repeat-containing protein 1 isoform 1 variant, partial	gi|62897087	66,870	6.17	254	1.65	1.05	1.64
240	LMNA	Lamin isoform E	gi|544063464	55,843	6.55	153	1.47	1.17	1.36
265	PKM2	Pyruvate kinase, muscle	gi|31416989	58,512	7.96	214	−4.38	−3.89	−5.13
275						213	−1.59	1.34	−1.36
267	PKM2	Chain A, Structure Of Human Muscle Pyruvate Kinase	gi|67464392	60,277	8.22	212	1.46	1.16	1.35
270	CCT5	T-complex protein 1 subunit epsilon isoform e	gi|807066366	55,770	5.33	116	2.22	1.38	1.85
288	CCT5	T-complex protein 1 subunit theta isoform 1	gi|48762932	60,153	5.42	133	1.73	1.21	1.37
324	G6PD	Glucose-6-phosphate dehydrogenase, isoform CRA_b	gi|119593089	59,467	8.27	166	1.71	1.51	1.37
325						74	−2.17	−2.50	−1.72
326						78	−1.68	−3.02	−2.23
388	PDIA5	Protein disulfide isomerase-related protein 5, partial	gi|1710248	46,512	4.95	136	−2.10	−1.36	−1.07
404	SERPINH1	Serpin peptidase inhibitor, clade H (heat shock protein 47), member 1, (collagen binding protein 1)	gi|47124471	46,559	8.75	106	2.25	1.11	1.37
405						111	3.08	1.08	1.68
408						112	5.17	1.25	2.42
505	PSAT1	Phosphoserine aminotransferase isoform 1	gi|17402893	40,796	7.56	181	−1.90	−1.06	−1.96
536	GAPDH/G3P	Glyceraldehyde-3-phosphate dehydrogenase isoform 2	gi|378404908	31,699	7.15	158	1.89	1.45	1.69
897	GAPDH/G3P	Glyceraldehyde-3-phosphate dehydrogenase	gi|31645	36,202	8.26	87	28.48	10.49	28.05
738	TAGLN	Transgelin, isoform CRA_c	gi|119587704	23,748	8.54	173	5.74	2.73	3.80
902	TAGLN	Transgelin	gi|48255905	22,653	8.87	153	1.76	1.35	1.61
750	TAGLN2	Transgelin-2 isoform a	gi|469608410	24,609	8.41	121	2.36	2.24	2.25
873	P4HB	Protein disulfide-isomerase precursor	gi|20070125	57,480	4.76	214	−1.51	−2.37	−1.22

^(a)^ Spot ID means numbers in 2-DE-images in Figure 1. ^(b)^ Acc. no. is a NCBInr database accession number and an entry of the UniProt/SWISS-PROT database. ^(c)^ The nominal mass is the integer mass of the most abundant naturally occurring stable isotope of an element. The nominal mass of a molecule is the sum of the nominal masses of the elements in its empirical formula. ^(d)^ Protein score is −10*Log (*p*), where *p* is the probability that the observed match is a random event. Protein scores greater than 62 are significant (*p* < 0.05). ^(e)^ NMX, normoxic condition; HPX6, hypoxic condition for 6 h; HPX12, hypoxic condition for 12 h; HPX24, hypoxic condition for 24 h.

## Data Availability

All relevant data can be found and are available within the manuscript and Supporting Information.

## Supplementary Materials

The following supporting information can be downloaded at: https://www.mdpi.com/article/10.3390/cells12242852/s1, Figure S1: Discovery of oxygen-tension-related additional factors in ASCs using proteomic analyses. Representative silver-stained 2-DE gel images of ASCs under normoxic environment; Table S1: List of identified additional proteins showing differential expression in hASCs according to the exposure time of the hypoxic environment; Table S2: Detectable adipokines using a human adipokine array kit.
