# Supplementary material for: Enhanced Osteocyte Differentiation: Cathepsin D and L Secretion by Human Adipose-Derived Mesenchymal Stem Cells
Source: Cells. 2023 Dec 17;12(24):2852. doi: 10.3390/cells12242852 (PMC10742070; doi:10.3390/cells12242852)

Supplementary Figure 1. Discovery of oxygen tension-related additional factors in ASCs using proteomic analyses. Representative silver-stained 2-DE gel images of ASCs under normoxic environment.

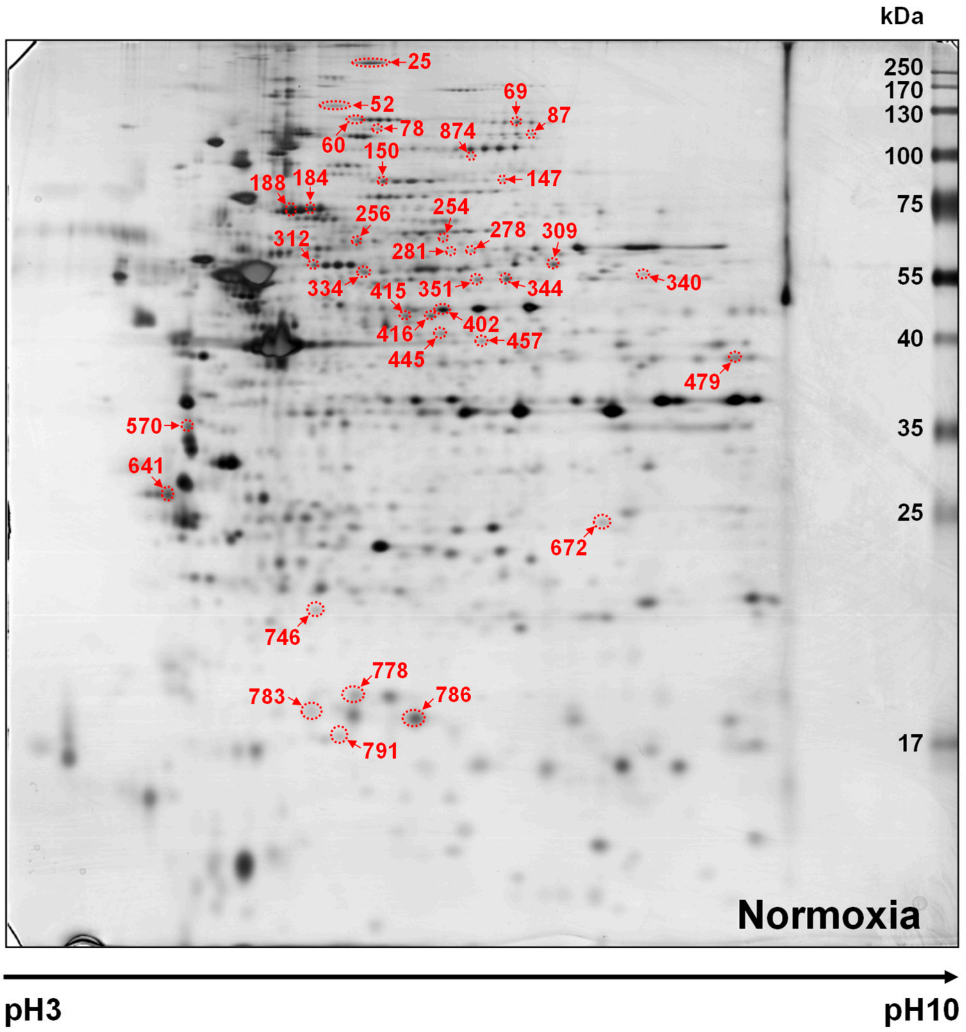

Supplement: Supplementary file 1 [file cells-12-02852-s001.zip › Supplementary Figure 1.pdf]
