# Supplementary material for: Enhanced Osteocyte Differentiation: Cathepsin D and L Secretion by Human Adipose-Derived Mesenchymal Stem Cells
Source: Cells. 2023 Dec 17;12(24):2852. doi: 10.3390/cells12242852 (PMC10742070; doi:10.3390/cells12242852)
Supplement: Supplementary file 1 [file cells-12-02852-s001.zip › Supplementary Table 1.pdf]

Supplementary Table 1. List of identified additional proteins showing differential expression in hASCs according to the exposure time of the hypoxic environment.

Spot ID [a)]	Gene name	Description	Acc. no.[b)]	Nominal mass (*Mr*) [c)]	Calculated PI	Score [d)]	Fold change		
							(H6/N) [e)]	(H12/N) [e)]	(H24/N) [e)]
25	TLN1	Talin	gi|6739602 [b)]	271653	5.77	171	-1.51	-1.23	-1.59
52	KRT9	Cytokeratin 9	gi|435476	62320	5.19	104	-1.73	-1.80	-3.87
60	VCL	Vinculin	gi|24657579	117234	5.83	179	-1.46	-1.20	-1.21
69	ACLY	ATP-citrate synthase isoform 2	gi|38569423	120608	6.95	200	-1.53	-1.06	-1.54
78	GANAB	Glucosidase, alpha; neutral AB, isoform CRA_a	gi|119594451	104930	5.85	128	-1.45	nd	nd
87	SND1	Chain A, Crystal Structure Of A Truncated Human Tudor-Sn	gi|197305029	64223	6.24	191	-1.78	-1.02	-2.11
147	SEC23A	Protein transport protein Sec23A	gi|38202214	87018	6.64	104	1.45	1.24	-1.22
184	ANXA6	Annexin A6 isoform 1	gi|71773329	76168	5.42	258	1.58	1.61	1.36
188	HSPA8	Heat shock cognate 71 kDa protein isoform 2	gi|24234686	53598	5.62	186	-1.59	-1.16	-1.59
254	USP14	Ubiquitin carboxyl-terminal hydrolase 14 isoform a	gi|4827050	56489	5.20	86	1.59	1.06	1.14
256	TCP1	T-complex polypeptide 1	gi|36796	60869	6.03	171	-1.46	-1.11	-1.50
278	YARS	Tyrosine--tRNA ligase, cytoplasmic	gi|4507947	59448	6.61	98	1.75	1.29	-1.02
281	YARS	Tyrosyl-tRNA synthetase variant, partial	gi|62898948	59467	6.79	83	1.76	1.25	-1.35
309	CAP1	Adenylyl cyclase-associated protein 1	gi|5453595	51926	8.07	148	-1.80	-1.36	-1.07
312	PDIA3	Protein disulfide isomerase family A, member 3, isoform CRA_a	gi|119597640	54454	6.78	158	1.76	1.87	2.10
334	KRT9	Keratin, type I cytoskeletal 9	gi|55956899	62255	5.14	79	-2.15	-1.80	-1.81

340	UGP2	UTP-glucose-1-phosphate uridylyltransferase (EC 2.7.7.9), skeletal muscle [validated]	gi|2136353	57101	8.43	71	-2.29	1.18	1.15
344	FSCN1	Fascin	gi|4507115	55123	6.84	141	1.54	1.18	-1.06
351	FSCN1	Fascin	gi|4507115	55123	6.84	117	1.60	1.32	1.17
402	ENO1	Enolase 1 variant, partial	gi|62896593	47453	7.01	127	1.47	1.10	1.27
415	EEF1G	Eukaryotic translation elongation factor 1 gamma, isoform CRA_a	gi|119594429	36554	5.21	106	1.53	1.06	-1.22
416	EEF1G	EEF1G protein, partial	gi|39644794	50156	6.27	97	1.65	1.17	1.23
457	KRT9	Keratin, type I cytoskeletal 9	gi|55956899	62255	5.14	74	2.30	1.17	-1.24
479	ALDOA	Fructose-bisphosphate aldolase A isoform 1	gi|4557305	39851	8.30	188	1.51	1.18	1.29
570	TPM1	Skeletal muscle tropomyosin, partial	gi|339958	26573	4.64	114	-1.52	-1.82	-2.39
641	YWHAE	14-3-3 protein epsilon	gi|5803225	29326	4.63	81	-1.71	-1.43	-1.25
672	EIF4H	Eukaryotic translation initiation factor 4H isoform 2	gi|14702180	25242	7.79	78	-1.69	1.12	-1.52
746	PRDX2	Peroxiredoxin-2	gi|32189392	22049	5.66	120	1.54	1.80	1.57
778	NME1	Nucleoside diphosphate kinase A	NDKA_HUMAN	17309	5.83	75	-1.50	-1.23	-1.37
783	SSR4	Translocon-associated protein subunit delta isoform 2 precursor	gi|5454090	19158	5.76	94	-1.45	1.17	-1.62
786	CFL1	Cofilin-1	COF1_HUMAN	18719	8.22	98	-1.61	-1.01	-1.23
791	STMN1	Stathmin 1 variant, partial	gi|62088144	15264	8.47	66	-1.54	-1.73	-1.69
874	PYGB	Glycogen phosphorylase B	gi|1172226	97306	6.26	231	-1.58	-1.47	-2.10

a) Spot ID means numbers is 2-DE-images in Supplementary Figure 1.
b) Acc. no. is a NCBInr database accession number and an entry of UniProt/SWISS-PROT database.
c) The nominal mass is the integer mass of the most abundant naturally occurring stable isotope of an element. The nominal mass of a molecule is the sum of the nominal masses of the elements in its empirical formula.
d) Protein score is -10*Log (P), where P is the probability that the observed match is a random event. Protein scores greater than 66 are significant (p<0.05).
e) H6, 12, and 24 means exposed hASCs under hypoxic conditions for 6, 12, 24 hrs, respectively. N means hASCs under normoxic environment. Fold changes are the values dividing the spot density of the hypoxic ASCS into the spot density of the normoxic ASCs.
