# Supplementary material for: Enhanced Osteocyte Differentiation: Cathepsin D and L Secretion by Human Adipose-Derived Mesenchymal Stem Cells
Source: Cells. 2023 Dec 17;12(24):2852. doi: 10.3390/cells12242852 (PMC10742070; doi:10.3390/cells12242852)
Supplement: Supplementary file 1 [file cells-12-02852-s001.zip › Supplementary Table 2.pdf]

Supplementary Table 2. Detectable adipokines using Human Adipokine Array Kit

Adiponectin	FGF basic	M-CSF
Angiopoietin-1	FGF-19	MIF
Angiopoietin-2	Fibrinogen	Myeloperoxidase
Angiopoietin-like 2	Growth Hormone	Nidogen-1/Entactin
Angiopoietin-like 3	HGF	Oncostatin M
BAFF/BLyS	ICAM-I	Pappalysin-1/PAPP-A
BMP-4	IGFBP-2	PBEF/Visfatin
Cathepsin D	IGFBP-3	PCSK9
Cathepsin L	IGFBP-4	Pentraxin-3
Cathepsin S	IGFBP-6	Pref-1/DLK-1/FA1
CCL2/MCP-1	IGFBP-rp1/IGFBP-7	RAGE
CCL5/RANTES	IL-1 beta	Resistin
Chemerin	IL-6	Serpin A12
Complement Factor D	IL-10	Serpin A8/AGT
C-Reactive Protein	IL-11	Serpin E1/PAI-1
CXCL8/IL-8	LAP (TGF-ß1)	TIMP-1
DPPIV/CD26	Leptin	TIMP-3
Endocan	LIF	TNF-alpha
EN-RAGE	Lipocalin-2/NGAL	VEGF
Fetuin B		
